# Experimental Characterization of the Mechanical Properties of Filter Media in Solid–Liquid Filtration Processes

**DOI:** 10.3390/ma17184578

**Published:** 2024-09-18

**Authors:** Vanessa Puderbach, Ralf Kirsch, Sergiy Antonyuk

**Affiliations:** 1 Institute of Particle Process Engineering, University of Kaiserslautern-Landau (RPTU), Gottlieb-Daimler-Strasse, 67663 Kaiserslautern, Germany; 2 Fraunhofer Institute for Industrial Mathematics (ITWM), Fraunhofer-Platz 1, 67663 Kaiserslautern, Germany

**Keywords:** filter medium deformation, nonwoven, mechanical testing, tension, viscoelasticity, aging

## Abstract

Nonwoven filter media are used in many industrial applications due to their high filtration efficiency and great variety of compositions and structures which can be produced by different processes. During filter operation in the separation process, the fluid flow exerts forces on the filter medium which leads to its deformation, and in extreme cases damage. In order to design or select a reliable filter medium for a given application, it is essential to have a comprehensive understanding of the mechanical properties of the nonwoven material. In general, the properties of the filter material are influenced by temperature and can be changed during loading due to irreversible deformation, fatigue, and aging processes. In order to gain a deeper comprehension, the presented study examines the influence of temperature and repeated tensile stress on the filter medium properties. The focus is on fuel and oil filters employed in automotive applications. The characteristic properties of the samples, including thickness, porosity, and permeability as well as Young’s modulus and Poisson’s number, are measured. Young’s modulus is determined for both new and aged samples. In addition, the viscoelastic behavior is investigated via a dynamic mechanical thermal analysis. The results demonstrate a significant dependence of mechanical properties on the material composition and the aging effects.

## 1. Introduction

During the filtration process, the flowing fluid applies a force on the filter medium, leading to its deformation. The extent of this deformation depends on the mechanical and microstructural properties of the filter medium and the operating conditions, such as flow rate, pressure, fluid viscosity, and temperature. This presents a challenge to filter designers who must minimize deformation in order to avoid undesirable effects like pleat collapse, pleat crowding, etc. [1]. Common countermeasures include the use of spacers [2], supporting ribs, and pleat holders. In order to fill a large filter area with nonwovens, stabilizing aids such as bulk fills or grids are used, which cause a reduction in the filter area as these stabilizers cover some of the pores of the filter media [3]. The requirement for an optimum filter design is therefore to find the right ratio of stability to filter area to ensure efficient filter operation. This optimal design is also dependent on the specific application and the particles to be separated, as well as on hygiene requirements. An empirical approach to determining the optimal design for stabilizing filter media can become very costly.

Nonwoven materials are used in many filtration processes, which is why they occupy a pivotal position in this study. The present study focuses on the liquid filtration process and especially the testing of oil and fuel filter media. The aim of this work is to find out which factors influence the characteristics and structural mechanical properties of nonwoven filter media and to what extent the effects of these influences can be predicted by models. 

### 1.1. Standard Tests for Characterizing Nonwovens

In the literature, the characterization of filter medium properties is described in a variety of studies that employ both experimental and also numerical investigations. 

A variety of methods may be employed to conduct experimental mechanical tests, including the utilization of diverse load modes, such as tension, compression, or bending. Furthermore, the number and frequency of loading cycles, or the test temperature, may be varied in order to gain a deeper insight into the material. The stiffness, elasticity, and tensile strength of nonwoven media are determined by the combination of the fiber and binder materials in the microstructure and manufacturing process [4,5,6,7]. Established test methods to determine the properties of nonwovens are presented in a series of ISO standards (ISO 9073-1 to ISO 9073-18) [1,2,3]. which are applicable to all kinds of nonwoven textiles. ISO 9073-1 [8] describes the measurement of mass per unit area, while the thickness measurement is addressed in ISO 9073-2 [9]. The tensile strength is outlined in ISO 9073-3 [10] while ISO 9073-15 [11] provides guidance on the measurement of air permeability. These properties yield a comprehensive characterization of the filter medium; however, when it is loaded with the filtration liquid (e.g., oil), the structural parameters, such as fiber composition and orientation, as well as the oil properties must be considered [12].

The fibrous material is composed of single fibers and may contain additives and binders [13,14,15]. The experimental test can be performed on a single fiber or on the fibrous filter medium.

### 1.2. Characterization of Single Fibers

The single fiber tests provide valuable insights into the deformation characteristics of the material from which the fibers are composed, while the distribution of their mechanical properties yields essential input data for the numerical modeling of fiber behavior. Single fiber tests deliver important information about the deformation behavior of the material of fibers, and the spread of their mechanical properties provides crucial parameters for the numerical modeling of fiber behavior [16,17].

The fibers of the nonwoven materials investigated in this study were composed of glass, polyester, cellulose or PET, and PBT. The density of cellulose fibers is typically within the range of 1.3 to 1.6 g/cm^3^ [18], while glass fibers exhibit a solid material density of approximately 2.5 g/cm^3^ [18,19]. Examples of the single fiber properties are provided by Bisanda et al. [20]. Pineapple leaf fibers, comprising 85% cellulose and displaying a density of 1440 kg/m^3^, have Young’s modulus ranging from 34 to 82 GPa. Satyanarayana et al. [21] listed the initial modulus of cotton, which has a high amount of cellulose (90 mass%) and a density of 1500 kg/m^3^, as 1.1 GPa. The glass fibers (with a density of 2540 kg/m^3^) have an initial modulus between 68 GPa and 96 GPa, while polyester resin has a density of 1300 kg/m^3^ and an initial modulus of 2.06 GPa [21]. 

### 1.3. Experimental and Numerical Testing of Nonwovens

The testing of the fibrous material delivers information about fiber interactions and the influence of fiber amount, additives, and other factors. 

The tensile tests conducted by Jubera et al. [22] on polypropylene nonwovens initially resulted in the damage of inter-fiber bonds, followed by a rearrangement of the fiber orientation. At high testing temperatures, the polypropylene nonwovens show a decrease in strength and a slight increase in energy absorption capacity [22]. Also, a study examining nonwoven felts composed of glass fibers revealed a fracture of the interbundle bonds, followed by fiber rearrangement prior to the total fracture of the specimen [5]. In situ tensile tests conducted in an X-ray micro-computed tomography (µCT) demonstrated fiber reorientation and fiber alignment when the tension increased. In some regions where the fibers were entangled, the interlocking structures exhibited enhanced strength [23].

Numerical image processing techniques allow the analysis of images obtained using scanning electron microscopy (SEM) or µCT facilitating the determination of the mean pore size of the porous structure [3,24,25,26,27,28,29,30,31], the computation of the fiber diameter distribution of the filter medium [32,33], and the analysis of the change in the microstructure occurring as a result of mechanical deformation [34].

The tensile strength of nonwovens can even be increased by notches and cracks [35]. 

However, due to the manufacturing process, nonwoven materials often have an anisotropic, mechanical behavior, which depends on the fiber orientation in the machine (md) and cross direction (cd) as well as thickness direction (zd) [36,37]. 

The manufacturing process parameters of needle-punched nonwoven geotextiles define their puncture resistance behavior, where there is an optimum level of punch density and depth that leads to the best puncture resistance of the nonwoven [38]. Other manufacturing parameters like feeder cylinder and doffer speeds of carding machines influence the thickness and tensile strength of the nonwoven where a higher feeder speed leads to an increase in thickness and an increase in toughness in the cross direction but also a decrease in tenacity in the machine direction [39].

### 1.4. Influence of Environmental Conditions on Filter Medium Properties

The mechanical behavior during the compression of geotextiles was described in the work by Kothari [40] depending on the thickness of the sample and the corresponding pressure during loading and unloading. Jaganathan et al. [41] showed an exponential decrease in the mean pore size in nonwoven fabrics with increasing applied pressure. The compression rate of a filter medium influences the dust-holding capacity by changing the mechanical structure of the filter medium [42,43]. 

Various models to describe the compression of a porous solid medium by a correlation of pressure and volume change can be found in Kawakita et al.’s study [44,45].

The question arises whether standard mechanical material testing under dry conditions is sufficient for a prediction of the filter media deformation caused by fluid flow and how the behavior changes due to aging of the filter medium. Aminu and Bahr [46] described the flow-induced deformation of the nonwoven wet electrospun polyacrylonitrile fiber mats that showed a nonlinear relationship between pressure and deformation. The variety of applications, for example, as geotextiles, in air purification, or as oil filtration requires the implementation of different tests to characterize the mechanical behavior in a given use case. The strength of biodegradable nonwoven wipes is significantly greater in a dry condition than in a wet condition for md and cd [47]. The principles of aging are presented in the standard 50035 [48]. This standard describes the aging of polymer materials which can be separated into inner and outer mechanisms. The inner mechanisms refer to the thermodynamic unstable conditions of the material itself with no external influences. In contrast, the outer mechanisms include chemical, physical, and microbiological influences on the material. For example, the nonwovens used for geotextile industries need a robust resistance to ultraviolet light (UV), due to their prolonged exposure to sunlight. It is also essential that medical devices exhibit UV stability when subjected to UV sterilization [49]. The physical changes by UV aging on polyethylene terephthalate (PET) nonwoven geotextiles show a decrease in tensile strength and no effect on the mass per unit area and thickness of the specimen [50]. The incorporation of additives during the formation of biopolymer (polylactic acid) fibers has been observed to alter the microstructure, thereby enhancing the UV stability of the material when the ceramic zinc oxide (ZnO) particles are introduced [49]. 

The thermal stability of polypropylene nonwoven can be increased by a modification with calcium carbonate (CaCO_3_), as well as the UV stability. However, the modification did not result in an increase in the acid corrosion resistance when subjected to higher acid conditions [51]. At higher temperatures, the polyester membrane undergoes a change in its polymeric structure, enabling the penetration of water into the polymeric matrix by means of chemical interactions [52]. Glass fiber polyester composites exhibit temperature-dependent behavior, displaying a substantial reduction in mechanical stability when subjected to temperatures exceeding 100 °C [53].

### 1.5. Numerical Modeling

Appropriate models and simulation tools can help reduce the need for testing prototypes. However, this requires the implementation of experimental methods to identify the relevant mechanical properties of nonwoven filter media and to validate the simulation. 

Numerical continuum models of polypropylene nonwovens have already been implemented in the Finite Element Method (FEM) for the simulation of tensile behavior [54,55]. The model is capable of reproducing the elasto-plastic behavior of the material for regular and notched specimens [54]. The anisotropy of thermally bonded nonwovens was predicted by numerical tools that implement the microstructure into the model by image processing of the µCT or SEM of the filter media [56]. The numerical computation methods represent also the failure patterns of the experimental tensile tests and can be used to analyze the various changes in the fiber network [57]. In contrast to a continuum model, the modeling of the actual fibers in a fibrous structure can consider the fiber material properties and structural characteristics, allowing the model to effectively predict the monotonic tensile behavior and the stress–strain behavior of the material at changing stresses [58,59]. Furthermore, the resolved flow simulations on the discrete filter media model can be employed to predict the permeability and particle separation [60,61,62,63].

This study examines the characteristics and mechanical properties of five filter medium materials, including thickness, mass per unit area, density, porosity, permeability, Young’s modulus, Poisson’s ratio, and compressibility. The materials are subjected to compression and tension tests to assess their mechanical behavior. Furthermore, aging tests in heated oil are performed in order to ascertain how a realistic material usage will affect the material properties.

## 2. Materials and Methods

A variety of different nonwoven filter media, provided by several manufacturers, is investigated in the study. The exact composition and manufacturing of the materials are confidential. The properties of the media (including thickness, fiber sizes, etc.) are determined in this study and presented in the results section. The first subsection presents the filter media materials and applications of the investigated samples. The investigational methods in the second subsection are divided into characteristic properties and structural–mechanical properties as well as aging behavior. 

### 2.1. Studied Filter Media

**Figure 1:**
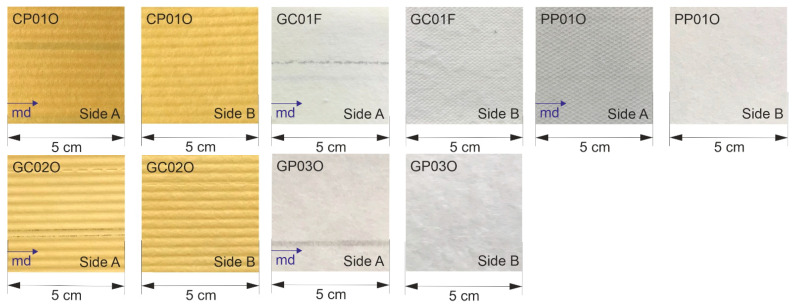
Photography of the filter medium samples.

The composition and application of the studied nonwoven filter materials are given in Table 1. Due to confidentiality, the identifiers given by the manufacturers are replaced by new sample names that include the fiber composition (Cellulose C, Glass G, and Polymer P) and numbering. The letter at the end describes the application, where F stands for fuel filtration and O for oil filtration (engine oil or automatic transmission fluid (ATF)). The samples are cut from a flat filter medium sheet and presented in Table 1 and Figure 1. The images show both sides A and B of each filter medium sample.

### 2.2. Characterization of the Filter Medium Properties

#### 2.2.1. Characteristic Microstructural Properties

The porosity, binder properties, and fiber structures are decisive factors in the properties of the porous medium. The filter medium samples are tested in standard test procedures to obtain the porous medium properties. 

First, the thickness $d_{S}$ of the filter material sample is determined, which is crucial to the later calculation of many material properties. In uniaxial compression tests according to ISO 9073-2 [9,64], the thickness of nonwoven textiles is determined using the Texture Analyser (TA, Stable Micro Systems, force range 0.01–500 N, resolution 10 mN, deformation rate 0.01–40 mm/s, displacement resolution 1 µm). A square filter medium sample of 4 cm edge length is placed on the horizontal fixed base plate of TA. The upper cylindrical steel punch with a diameter of 37 mm moves toward the sample at a rate of $v_{P}$ = 0.05 mm/s measuring force and displacement. When the pressure reaches the value of 0.5 Pa, the movement of the probe is stopped. After a holding time of 10 s during which the force is kept constant, the thickness of the sample between the probe and the base plate is measured according to ISO 9073-2 [9]. The average thickness of each material was measured with three samples. 

The mass per unit area $m_{A}$ is an often-used parameter for the description of the filter material. It is determined by weighing the samples with a sample surface area A = 36 cm^2^ using a lab scale (Chyo Balance Corp. with a resolution of 0.1 mg, Tokyo, Japan) according to ISO 9073-1 [8]. Afterwards, these samples are used for the measurement of the solid density and porosity by helium pycnometry (Ultrapycnometer 1000, Quantachrome, Boynton Beach, FL, USA). The measurement determines the void fraction of the material samples. Two samples of each material are measured 10 times in a row. 

The sample porosity ε is defined as the ratio between the void fraction $V_{V}$ and the total sample volume $V=A\cdot d_{S}$ as follows [65]:

(1) $$\varepsilon =\frac{V_{V}}{V}=1-\frac{V_{S}}{V}$$

where $V_{S}$ is the solid volume fraction.

The solids density $\rho_{S}$ of the porous sample is calculated from the results of the pycnometry. It is defined as the ratio between m and $V_{S}:$

(2) $$\rho_{S}=\frac{m}{V_{S}}$$

The permeability of the filter medium is described by the flow velocity $\bar{v}$ of the air stream passing the sample at a defined differential pressure [34]. The permeability gives information about the resistance to flow through the porous filter medium of a filter medium and is an important parameter in order to compare filter materials by their pressure drop. It is measured according to DIN EN ISO 9237 [66] with the FX 3300 LabAir IV (TEXTEST Instruments, Schwerzenbach, Switzerland). The differential pressure of Δp = 200 Pa is applied at eight different positions with the circular measuring area of $A_{P}$ = 20 cm^2^ on each sample. The Darcy equation [67,68] describes the pressure drop in relation to the medium thickness with the permeability B (in m^2^):

(3) $$B=\frac{{\bar{v}\cdot d}_{S}\cdot \eta }{\Delta p}$$

where Δp is the differential pressure, $\bar{v}\;$ is the flow velocity, $d_{s}$ is the thickness of the filter medium, and η describes the dynamic viscosity. 

#### 2.2.2. Compression Test

The compression tests are performed using the TA where the sample is compressed with the flat steel punch. The test velocity or strain rate v of loading and unloading is 0.05 mm/s. The sample is loaded up to a maximum force of 50 N and unloaded five times to investigate the cyclic compression behavior. The time of 70 s is held between individual loading–unloading cycles for possible relaxation. The axial deformation is measured as the displacement of the punch compared to the base plate. The force-displacement data are used to calculate the pressure-density relationship of the material. 

The compressibility of a material describes the relative volume reduction that occurs due to an increase in pressure, as defined by Equation (4). The density of a porous material is defined as the mass per unit area divided by the thickness of the sample $\rho \left(t\right)=m_{A}/\left(A୿z\left(t\right)\right).$ In this context, a constant area is assumed when the pressure p=F/A is calculated. Equation (5) describes the compressibility index using a model proposed by Johanson [42,44,69,70,71].

(4) $$\kappa =\frac{dV/V_{0}}{dp}$$

(5) $$\frac{\rho_{porous}}{\rho^{*}}={\left(\frac{p}{p^{*}}\right)}^{n}$$

The compressibility index n is calculated from the slope of the linear fitting in a logarithmic scale of the density $\rho_{porous}$ and the pressure p, with a reference density $\rho^{*}$ and a reference pressure $p^{*}$. A material with n = 0 is an ideal incompressible such as water. Fine sand shows low compressibility with n = 0.01 up to 0.05. A value of n = 0.1 up to 1 indicates highly compressible behavior comparable to that of moist powders [69].

#### 2.2.3. Tensile Tests

**Figure 2:**
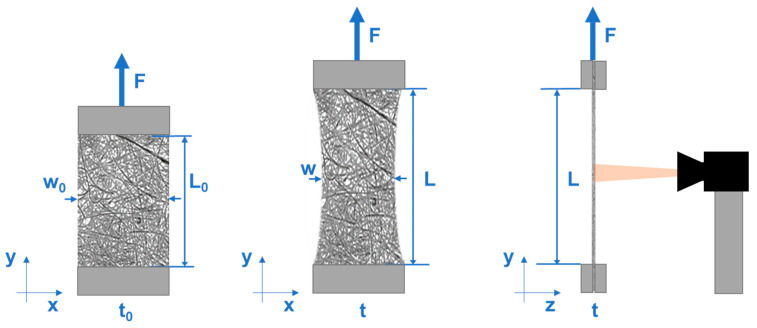
Tensile test scheme with optical measurement.

The tensile deformation behavior of the filter media is studied by a uniaxial tensile test that is shown schematically in Figure 2. The porous filter medium samples are fixed between two clamps, whereby the lower clamp is fixed, and the upper clamp moves upwards in the pulling direction during the test. The samples are prepared in md and in cd and all the tensile tests are performed in both directions to obtain the material anisotropy. The tensile tests are used to determine Young’s modulus, Poisson’s number [72], complex modulus, storage modulus, and loss modulus at different temperatures as well as the aging influence on Young’s modulus.

##### Determination of Young’s Modulus and Poisson’s Number

In the tensile tests conducted using TA, a camera (Basler ace 2 a2A2590-60ucBAS, Ahrensburg, Germany) was utilized to capture the lateral deformation, facilitating the calculation of the Poisson’s number. The test setup is shown schematically in Figure 2.

The filter medium sample is stressed once at room temperature with a maximum force of up to 50 N. The elongation, the lateral deformation, and the force are measured. Table 2 shows the test parameters of the tensile tests. 

**Figure 3:**
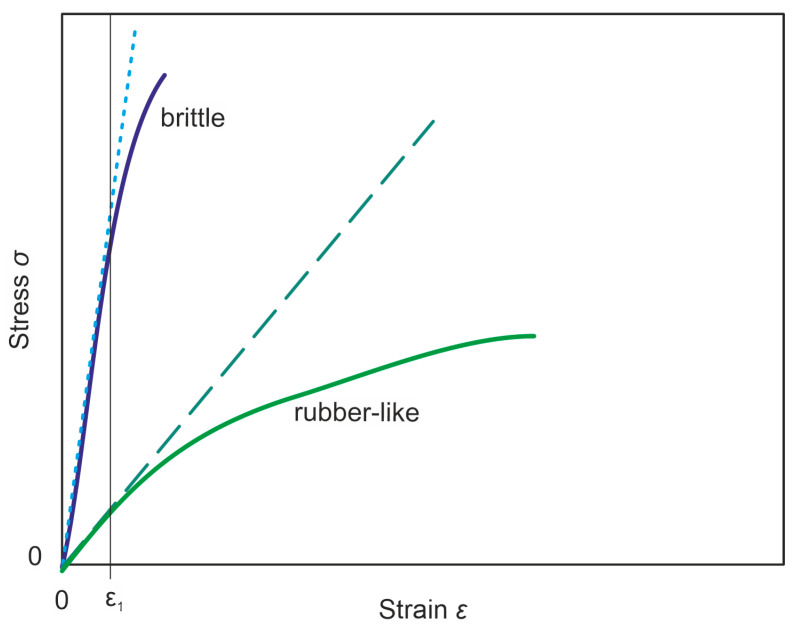
Schematic tensile behavior of different polymer types according to EN ISO 527-1 [72]. $\varepsilon_{1}$ marks the linear section of the curves, where the linear regression (dashed/dotted lines) can be applied.

The force-displacement results from the tensile tests are transformed into the stress–strain diagram $\sigma (\varepsilon_{L})$ for the calculation of $Y^{*}$. Figure 3 shows an example of brittle and rubber-like polymer tensile behavior [72]. The linear slope at the outset of the loading is described by Hooke’s law in Equation (6) [73] and used to obtain Young’s modulus $Y^{*}$:

(6) $$\sigma =\frac{F}{A^{*}}=Y^{*}\cdot \varepsilon_{L}$$

where $A^{*}$ is the sample cross-sectional area, and $\varepsilon_{L}=\Delta L/L_{0}$ represents the tensile strain of the sample with the initial length $L_{0}$ and the elongation ΔL.

The transverse deformation $\varepsilon_{t}=\Delta w/w_{0}$ is calculated from the changes in width Δw and the initial width $w_{0}$ which are then used to determine the Poisson’s number ν:

(7) $$\nu =-\frac{\varepsilon_{t}}{\varepsilon_{L}}$$

##### Determination of Complex Modulus at Different Temperatures

The repeated stress and high temperatures at operating conditions of an oil filter in its application influence the mechanical behavior of the filter medium. The high-frequency dynamic mechanical thermal analysis (DMTA, Netzsch Gabo Instruments EPLEXOR 500 N, Selb, Germany, max. load: 1500 N (static), 500 N (dynamic), max. dynamic elongation: 4 mm, frequency range: 0.01 Hz–100 Hz, temperature range: −150 °C up to 500 °C) investigates the elastic and viscous behavior of the samples at different temperatures and different frequencies. It is performed at 20 °C and at 60 °C for fuel filter sample GC01F and 100 °C for oil filter samples CP01O and GP03O. The elevated temperature is selected so that it corresponds to the temperature of the material in its typical application. The frequency range varies from 0.5 Hz up to 50 Hz with 15 logarithmically distributed values. The detailed test parameters are listed in Table 3. The test is repeated with five samples in each material direction.

**Figure 4:**
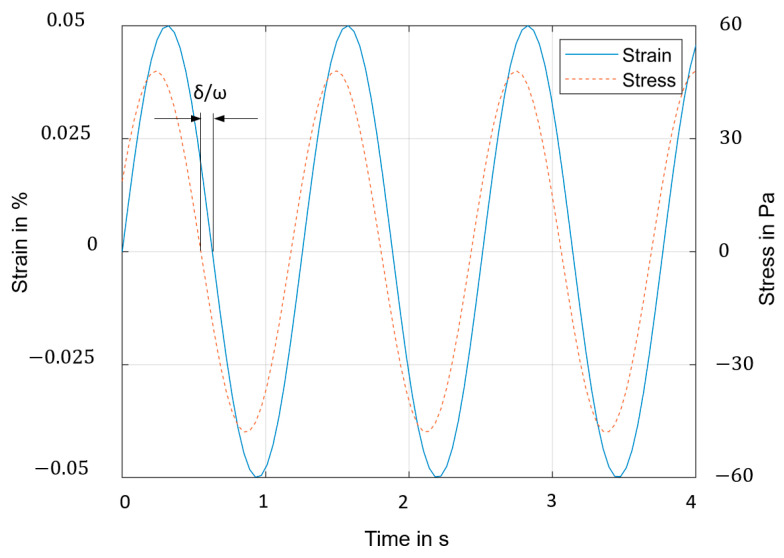
Typical stress–strain oscillation shift by a cyclic DMTA test of a viscoelastic material.

The maximum stress results from the cross-sectional area (thickness × width) of the sample and the maximum force (Equation (5)). With the defined maximum stress, the results of the time-dependent changes in the strain is measured. The stress and the strain curves of a viscoelastic material have the same frequency but different phasing δ/ω, where ω is the frequency of strain oscillations and δ the phase angle. Figure 4 shows the typical stress–strain curves of a cyclic test with viscoelastic material. The maximum cyclic strain is 0.05% to stay in the region of elastic material behavior.

The viscoelastic behavior is investigated by DMTA. It is described by the complex modulus $E^{*}$, that is composed of the elastic part of the deformation, the storage modulus $E^{/}$ and the viscous part of the deformation, the loss modulus $E^{//}$ [74,75]:

(8) $${E^{*}=E}^{/}+iE^{//}=\frac{\sigma_{0}}{\varepsilon_{0}}(cos\delta +sin\delta )$$

where $\varepsilon_{0}$ is the strain, $\sigma_{0}$ is the stress, and δ is the phase angle.

(9) $$tan\delta =\frac{E^{//}}{E^{/}}$$

##### Determination of Aging Behavior

In addition to repeated stress and high temperatures, environmental influences like aging of an oil filter can also influence the mechanical behavior of the filter medium.

The aging of polymer materials can be divided into two groups: inner aging and outer aging as explained in the introduction. The inner aging is investigated by tensile tests after one year of storage at room temperature. The outer aging is an effect induced by environmental conditions. To investigate the influence of oil wetting and temperature, the samples GC01F, GP03O, and PP01O are stored in heated engine oil (10W-40) at a temperature of 130 °C for 504 h. After storage, the warm samples are cooled down to room temperature and wiped off carefully to remove the excess oil. Afterward, the thickness and Young’s modulus of the aged samples are determined. The tests are performed with three samples each in md and cd. The change in the values Δx are calculated as follows:

(10) $$\Delta x=\frac{aged\;value-initial\;value}{initial\;value}\cdot 100\%$$

The thickness and Young’s modulus are compared before and after aging.

## 3. Results

In this section, the results of the previously described testing methods are presented. The comparison of the tensile results and the changes due to aging or cyclic testing are discussed in Section 4.

### 3.1. Dry Sample Microstructural Property Characterization

The microstructure defines the fluid–dynamical properties of the medium. The number of layers, thickness, porosity, and permeability as well as the structure of fibers in combination with binders or fiber entangling influences the pressure drop and filtration efficiency of a filter medium. 

**Figure 5:**
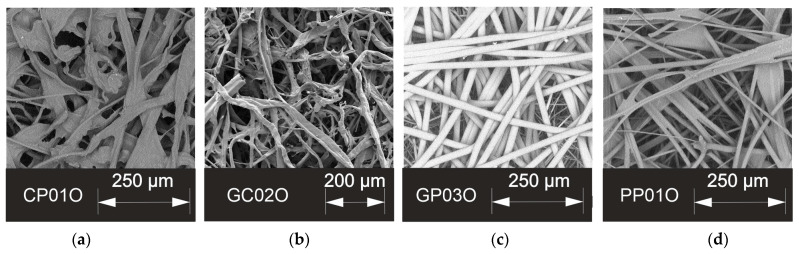
SEM images of the nonwoven surface containing cellulose and polymer fibers (**a**), glass and cellulose fibers (**b**), glass and polymer fibers (**c**), and only polymer fibers (**d**).

The SEM of the different samples gives an impression of the nonwoven surface of the oil filter medium samples and how the fibers and pores are arranged (Figure 5).

Cellulose fibers have an irregular shape; images a and b show high cellulose content of the nonwoven. Polymer and glass fibers have a rather round cross-section. But in the SEM images, it is not possible to differentiate between glass and polymer fibers. 

**Figure 6:**
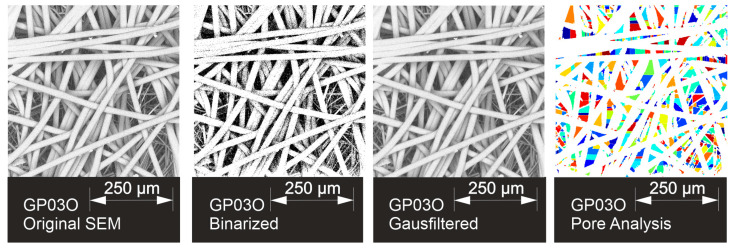
Image processing of GP03O SEM image, different colors in pore analysis mark different pores.

The image processing (MATLAB Image Processing Toolbox, Version R2024a, MathWorks Inc., Natick, MA, USA [29,32]) is demonstrated in Figure 6. The original SEM is binarized with a sensitivity of 0.8, and a Gaussian filter with a standard deviation of 1 blurs the image and is necessary for better identification of the single fibers. The pores are shown in different colors. The resulting distributions are shown in Table 4. 

The results of the thickness $d_{S}$, mass per unit area $m_{A}$, solid density $\rho_{S}$, and porosity ε testing are shown in Table 5. The fuel filter GC01F shows the largest values of $m_{A}$, while, at the same time, the lowest ε is much smaller than the values found for the other three samples that are oil filter medium samples; therefore, they have different requirements for use. 

The results of the permeability tests are listed in Table 6. It shows the air velocity and the air permeability according to Darcy (Equation (3)).

### 3.2. Compressibility

**Figure 7:**
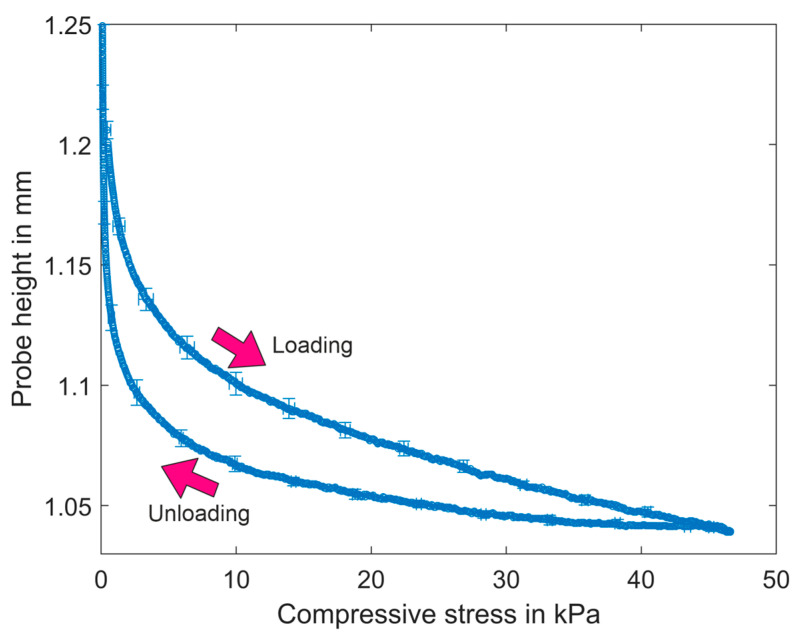
Compression curve for the first cycle of loading–unloading tests with material CP01O.

**Figure 8:**
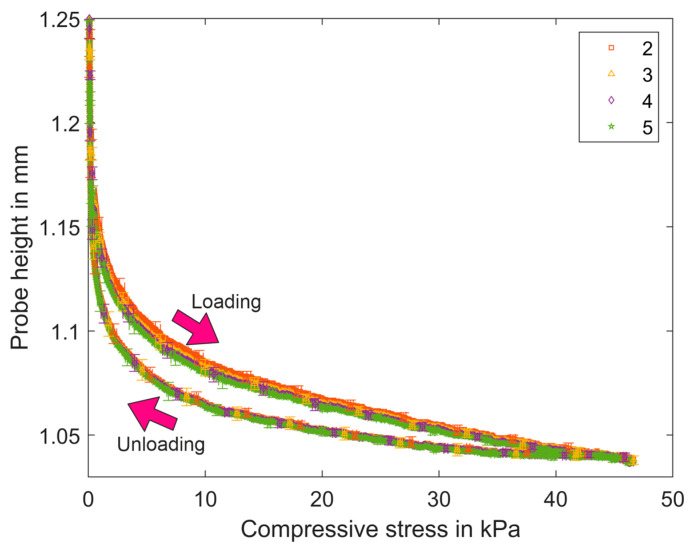
Compression curves measured by four repetitions of loading–unloading cycles of CP01O.

The compression tests are performed using filter medium material CP01O. A sample was loaded and unloaded five times up to a pressure level of 47 kPa. The results of the first cycle and of the four subsequent repetitions are shown in Figure 7 and Figure 8, respectively. The detailed values are given in Table 7. The standard deviation is displayed for every 50th value. The probe height at the point of maximum stress remains relatively unchanged across all cycles. The thickness of the samples was determined at 500 Pa; therefore, the initial value is simplified here and assumed at a pressure of 500 Pa. After the first cycle, the initial thickness of the sample was not reached. The plastic volume reduction is 5%, and subsequent repetitions result in a reduction in thickness that is less pronounced. The plastic volume reduction decreases from 2.6% to 2%.

The volume reduction in each cycle represents the difference between the volume before compression (initial volume of each cycle) and after compression relative to the initial volume of each cycle. This value is divided into two components: a plastic component, representing the volume reduction after compression, and an elastic component, representing the volume recovered after unloading. The viscous strain is represented by the volume component that can be regained after the waiting time until the next cycle starts. 

As illustrated in Table 7 and Figure 7 and Figure 8, the first cycle differs a lot from the following cycles. In the first cycle, the structure undergoes substantial plastic deformation, subsequently exhibiting elastic–plastic behavior with the plastic strain, which is reduced in the subsequent cycles. In the next cycles, the structure behaves elastically plastically with a viscous component, and saturation occurs in all deformation components as the number of cycles increases.

The compressibility is compared at the maximum pressure of 46 kPa in each cycle. Table 8 lists the compressibility and compressibility index of oil filter sample CP01O at 46 kPa according to Equations (4) and (5).

The loading curves indicate a reduction in probe height from the first cycle to the last cycle, with notable differences between the first loading and subsequent repetitions. The unloading curves are all similar. The compressibility and compressibility index decreased. There is a notable disparity between the initial loading and the subsequent loading, yet the subsequent reduction is only modest. These observations illustrate that the filter medium exhibits low compressibility characteristics.

### 3.3. Tensile Behavior

**Figure 9:**
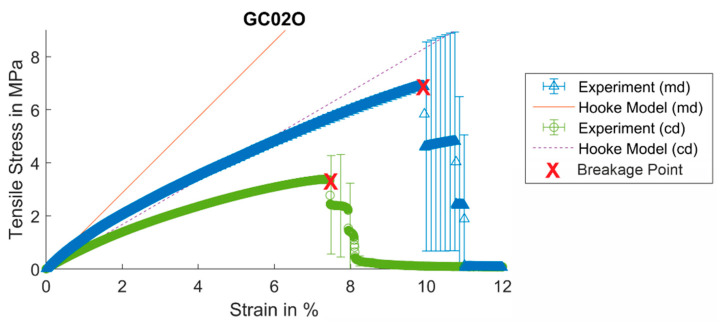
Mean stress–strain curve from the tensile test with GC02O with linear regression to calculate Young’s modulus.

The samples are analyzed in a tensile test as described in Section 2.2.3. The typical stress–strain-curve of the tensile test with filter medium material CP01O (md) is shown in Figure 9. The mean value with the corresponding standard deviation (every 50th value) is compared to the linear regression used for the calculation of Young’s modulus. Young’s modulus is calculated from the elastic deformation range at the beginning of the load that is characterized by the linear slope of the loading curve according to Equation (5). 

Young’s modulus $Y^{*}$ is obtained according to Hooke’s law in Equation (4) as the slope of the linear approximation in the elastic deformation. Young’s modulus in md is 142.88 MPa and cd is 83.48 MPa. The overview of all the tensile test results is presented in Table 9. The md sample of GP02O breaks at 6.89 MPa tension, and in contrast, the cd sample breaks at a lower tensile stress of 3.38 MPa. All md samples have a higher breaking resistance than the cd samples. The difference in breaking resistance and Young’s modulus results from the manufacturing process. Every filter medium sample is made from fibers and additives and the direction of the machinery has a major influence on the mechanical structure of the material. 

In the case of GP03O, the material is stressed but a breakage could not be reached in md. Young’s modulus in this case cd has a great standard deviation which could be explained by the inhomogeneous structure in correlation with the small medium samples for testing. The samples are clamped in place, this may have caused damage, or the sample may have slipped unnoticed in the holder clamps. 

Poisson’s number is calculated from the transverse deformation according to Equation (6). The deformation is really small; therefore, the maximum possible deformation is used for Poisson’s number calculation. In the cases of GP03O and PP01O, there was no transverse deformation observable. Table 10 shows the observed results of Poisson’s number at maximum deformation.

Poisson’s number of fuel filter sample GC01F is independent of direction, even though Young’s modulus and breaking strength are highly anisotropic. The oil filter samples CP01O and GC02O show a higher Poisson’s number in md. 

**Figure 10:**
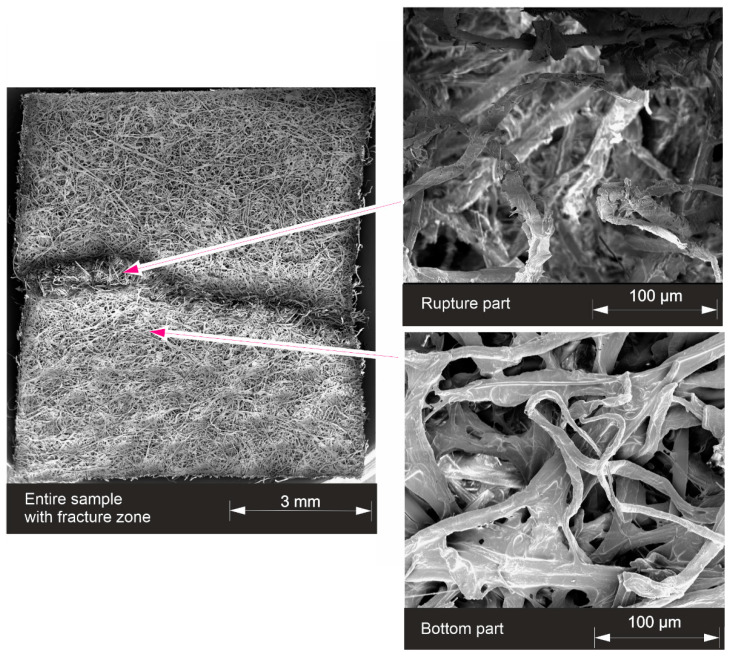
Broken sample GC02O.

**Figure 11:**
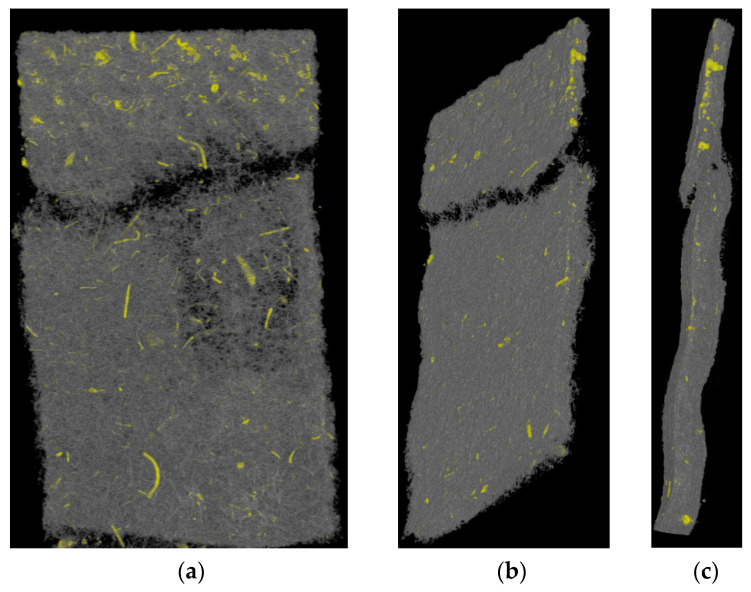
µCT images of oil filter sample GC02O; glass fibers are marked yellow. (**a**) top view (**b**) angled view (**c**) side view.

A sample of material GC02O is tested with TA until breakage and after this, it is investigated with SEM (Figure 10) and µCT (Figure 11) to figure out the structure of the rupture (For a comparison the unstressed structure of the material is shown in Figure 5). The entire broken sample is shown in addition to the closer look at the rupture part, where the fibers of the porous structure are pulled apart, as well as a closer look at the bottom part under the rupture. Due to the random alignment of the fibers in the nonwoven, a tearing edge is often unique and not always straight. 

The tearing edge of this glass and cellulose material shows a wavy shape. A constriction is clearly visible, which is necessary for determining Poisson’s ratio. The undamaged part of the structure (bottom part) does not show a difference from the unstressed sample in Figure 5. The images show flat and irregularly shaped fibers, which are cellulose fibers, and the glass content seems to be small in this material. The glass fiber content is analyzed further by µCT.

The µCT images reveal the wavy structure of this oil filter sample (Figure 11b,c). Additionally, by analyzing the gray scale values of the fibers, the glass fibers are marked yellow.

#### 3.3.1. Aging of the Samples

Due to the aging process performed in oil (described in Section 2.2.3 Determination of Aging behavior) with a temperature of 130 °C, the thickness of the material increased. The thickness was measured after cooling and is given in Table 11. The aged thickness is then used for the calculation of $Y^{*}$ from the tensile tests performed with aged samples. 

**Figure 12:**
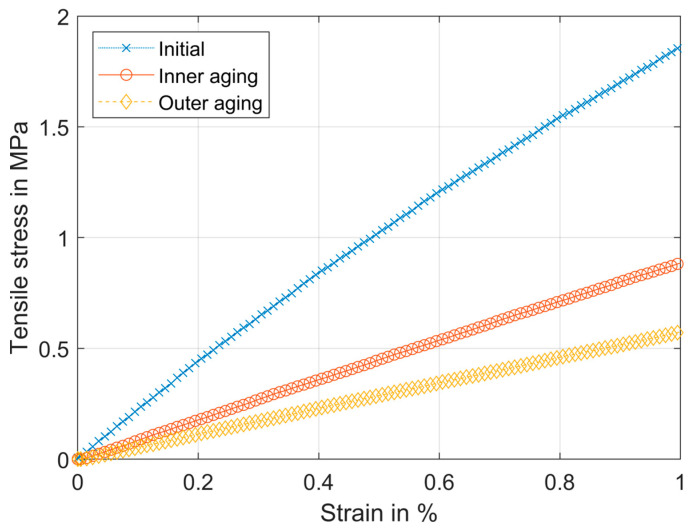
Typical stress–strain curve of GC01F md, initial state, after inner aging and after outer aging.

Figure 12 shows the changes in the porous structure by aging influences. Inner aging leads to a decrease in $Y^{*}$, which is represented by the slope of the curves. The outer aging by heated oil leads to a further decrease in $Y^{*}$ where the changes in thickness are considered. 

The measured values of $Y^{*}$ after aging are summarized in Table 12. The inner aging is the change after one year of storage and no external influences. Outer aging values are the changes values after three weeks of storage in heated oil at 130 °C. Both tests are carried out for the same aging time period. Therefore, strictly speaking, the external value also takes inner aging into account. The aging caused a softening of the materials.

It can be seen that inner aging has a significant influence on the material in the cases of GC01F and PP01O. To compare the values with the initial values, the deviation is calculated as a percentage according to Equation (9). The results are shown in Table 13.

The comparison shows that the aging has a major influence on the mechanical stability of the materials. In the case of GP03O, the inner aging shows an increase in Young’s modulus in cd, which is completely different from all other values that show a decrease. The initial values of the cd test have a great standard deviation, which leads to the conclusion that in this case, a measuring error leads to this unusual behavior. In the md case of GP03O, the inner aging is negligible. In general, the influence of outer aging in oil leads to a great decrease in Young’s modulus where the influence is more significant in md than in cd. 

#### 3.3.2. DMTA Cyclic Test Results

**Figure 13:**
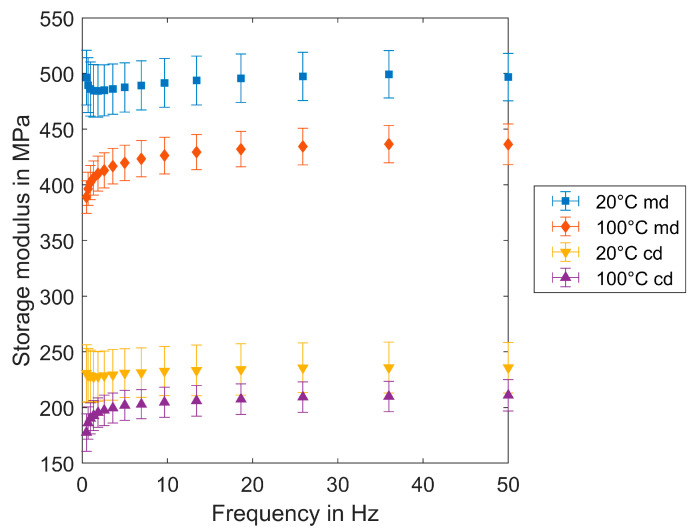
Storage modulus $E^{\prime }$ at 20 °C and 100 °C for the filter medium sample (CP01O).

The DMTA cyclic tests of filter medium samples are performed with small maximum strains (0.05%), where the stress–strain behavior is linear elastic as was shown with the tensile test performed with TA. Three different material samples are tested in cd and in md: fuel filter sample GC01F and oil filter samples CP01O and GP03O. The strain frequency varies from 0.5 to 50 Hz. The storage modulus of CP01O is shown in Figure 13.

The comparison between 20 °C and 100 °C for both md and cd delivers higher values at 20 °C. The frequency dependency at 20 °C is nearly constant; at 100 °C, there is an increasing trend; at low frequencies, the storage modulus increases until a constant value is reached at about 10 Hz. Comparing the values of md and cd, the values of md are about twice as high as those of cd.

**Figure 14:**
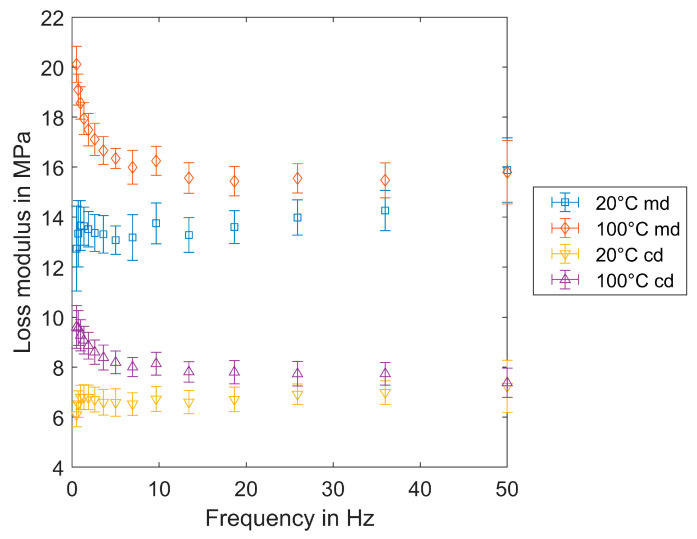
Loss modulus $E^{\prime \prime }$ at 20 °C and 100 °C (CP01O).

The loss modulus of CP01O (Figure 14) in md is also markedly higher than in cd. The temperature influence shows similar behavior in both md and cd. The loss modulus is significantly smaller than the storage modulus so the influence of temperature on the loss modulus is more significant. At low frequencies, the curves demonstrate a dependence on temperature, with a convergence of the curves as the temperature rises. At 50 Hz, both curves reach the same value. At 20 °C, the values reach 14 MPa (md) and 7 MPa (cd). At 100 °C in md, the loss modulus decreases from 20 MPa and reaches 16 MPa (md), while in cd, it is only half as large and decreases from 10 MPa until at 50 Hz in which it reaches about 8 MPa. 

The storage modulus is defined as the difference between complex and loss modulus; the loss modulus of measured filter media in the range of 0.5 Hz to 50 Hz is really small compared to the complex modulus, so the main behavior is defined by the storage modulus, which defines the elastic properties. The strain is really small for investigating the elastic behavior in the mainly elastic region, so it is not surprising that the behavior is dominantly elastic. The loss modulus at high temperatures is decreasing; it describes the viscous effects. 

The detailed results are listed in Table 14. Here, the mean value over all frequencies is shown for every material sample. The storage modulus describes the elastic deformation and is much higher than the loss modulus that describes the viscous effects, which leads to small phase angles. Those results show a viscoelastic behavior of the samples with a dominant elastic deformation, in all cases. In view of the influence of the temperature, only GC01F has temperature-independent behavior (in this case, the high temperature is only 60 °C). 

## 4. Discussion

The characterization of the dry samples provides insight into the composition and microstructure of the tested materials. Different samples are used, which are also applied in different filtration applications. Nevertheless, the SEM images and the test results provide a good understanding of the composition of the samples. The SEM images enable an analysis of the fiber diameters and pore sizes. It should be noted that only the fibers visible from the surface of the 2D image can be considered. Pycnometry determines the void volume fraction in the samples. As shown in Table 5, the samples with the largest void volume have the lowest mass per unit area (if the thickness is comparable). Different solid densities for the same porosity will also result in different values. The results of the solid density are in good agreement with values from the literature of the single fiber material of 1300 up to 1600 kg/m^3^ (cellulose) and 2500 kg/m^3^ (glass) [18,19,21]. The high value of the material CP03O of 1925 kg/m^3^ suggests that a very high amount of glass fibers is present. In contrast to the values for materials GC01F and GC02O, which are in the cellulose range and therefore presumably have a high cellulose content that could also be confirmed by the µCT analysis of GC02O. The results of the permeability measurement describe the flow of air through the filter medium. The results play a major role in providing a good overview of the properties of the filter media. Even if the porosity of the filter media is in a similar range, the material GC01F shows a much lower permeability than all other materials tested. Further analysis of the pore and fiber structure is required to ascertain the inter-relationships.

According to the compressibility index, the filter sample shows a low compressibility comparable to fine sand [69].

Young’s modulus of the porous fiber nonwoven is significantly lower than that of comparable single fibers. This is due to the nonwoven’s reliance on inter-fiber connections, including binder and weaving or entangling, as well as the influence of fiber content, including material and number of contacts. For example, cellulose fibers are in a range between 34 and 82 GPa [20,21]; nonwovens containing cellulose show values between 61 and 220 MPa. 

The aging tests show that the material properties of some materials are also time-dependent and change by storage only. The thickness of the glass–cellulose, glass–polymer, and polymer materials increases when it is in contact with heated oil for three weeks. The change in thickness also influences Young’s modulus. The outer aging referring to the usage parameters of oil filters shows a great change in material properties. In all test cases, the material’s Young’s moduli are almost halved after the storage of the material in heated oil. It confirms the results from the literature, which shows that mechanical stability decreases when glass fiber polyester composites are treated above 100 °C [53].

The high-frequency test also shows temperature dependency. The influence is relatively low, but all the materials have a slightly decreasing complex modulus with an increase in temperature. In general, the storage and loss modulus in md is higher than in cd, which can be confirmed by the results from TA testing, where Young’s modulus is also higher in md than in cd. This makes the anisotropy of the nonwoven a key parameter that is defined by the manufacturing process. 

The storage modulus is considerably higher than Young’s modulus. For instance, for material CP01O, $E^{\prime }$ in md is 490.9 MPa, while $Y^{*}$ is 143 MPa. This can be attributed to the significant difference in strain rate. The temperature-induced decline in values at elevated temperatures is corroborated by the aging test. 

## 5. Conclusions

In this work, the characteristics and properties of different filter media were experimentally determined in order to describe their mechanical behavior during operation. As many properties of the samples as possible were examined in order to determine the characteristic values of the samples. In this way, the properties of samples with similar characteristic values can be compared in the future. The measured characteristic values of fiber diameter distribution, thickness, mass per unit area, porosity, solid density, and air permeability represent the porous structure. The mechanical tests revealed the samples’ behavior under stress in an initial state and again after aging. The aging tests prove that the use of the filter material (which means oil contact at elevated temperatures) has a clear influence on Young’s modulus in most of the cases and leads to a decrease of 43% to 73%. It changes because of inner and outer mechanisms. Therefore, when utilizing the mechanical properties as parameters in a numerical simulation, it is essential to consider the alterations in thickness due to oil contact and the reduction in mechanical properties at elevated temperatures. The anisotropy of these fibrous filter medium samples is proven by tensile tests. The samples show higher tensile strength and higher Young’s moduli in the machine direction. The DMTA test shows that a higher temperature leads to a lower storage modulus in every test case and viscoelastic material behavior. 

The assumption made in this work that the filter material is homogeneous allows the mechanical values and anisotropy to be determined from measurements according to the laws of continuum mechanics. The results of the systematic investigations are used to validate the FEM simulations of the filter media and to couple the mechanical models with flow models in order to investigate the flow-induced deformations. Furthermore, the data obtained can be used for machine learning training. However, this assumption limits the influence of the microstructure, and the fiber materials used on the overall deformation.

Further investigations will investigate the flow-induced behavior of the filter media experimentally and numerically. The results of the mechanical parameters in this study will help improve numerical models because dry parameters might not be applicable for liquid filtration simulation. So, the parameters obtained in this study can become key parameters of flow-induced deformation simulations. 

For further investigations, it will be interesting to find out the influence of the manufacturing process and binder usage on the porous material structure and inter-fiber connections of the same fiber material, which is the most important characteristic defining the mechanical stability of a nonwoven. Considering this behavior is necessary for the 3D modeling of nonwoven deformation to optimize the performance.

In subsequent investigations, the applicability of these results in relation to numerical investigations of the flow through the filter media will be examined.

## Figures and Tables

**Table 1:** Studied filter medium materials.

Sample	Material Composition	Application
CP01O	cellulose, polyester	engine oil filtration
GC01F	cellulose, glass	fuel filtration
GC02O	cellulose, glass	engine oil filtration
GP03O	glass, polyester (layers), acrylic binder	ATF, engine oil filtration
PP01O	polybutylene terephthalate(PBT) (lattice structure), polyethylene terephthalate (PET)	oil filtration

**Table 2:** Parameters of tensile tests with TA.

Parameter	Unit	Value
Sample size (length × width)	mm × mm	50 × 24
Test length (initial clamp distance)	mm	20
Test velocity	mm/s	0.05

**Table 3:** DMTA parameters.

Parameter	Unit	Value
Sample size (length × width)	mm × mm	60 × 10
Test length (initial clamp distance)	mm	40
Maximum force $F_{max}$	N	5

**Table 4:** Properties of filter media from image analysis.

Sample	Mean Fiber Diameter in µm	Mean Pore Size in µm
CP01O	12.5 ± 4.6	4.3 ± 3.8
GC02O	10.1 ± 2.9	2.4 ± 2.7
GP03O	13.1 ± 4.0	4.3 ± 3.2
PP01O	10.3 ± 3.3	4.4 ± 3.7

**Table 5:** Characteristic properties of the filter media.

Sample	Thickness $d_{S}$ in mm	Mass per Unit Area $m_{A}$ in kg/m^2^	Solid Density $\rho_{S}$ in kg/m^3^	Porosity ε (d-)
CP01O	1.25 ± 0.01	221.0 ± 8·10^6^	1571 ± 24	0.89 ± 0.002
GC01F	0.95 ± 0.02	284.3 ± 5·10^6^	1559 ± 8	0.81 ± 0.001
GC02O	1.26 ± 0.01	219.6 ± 3·10^6^	1629 ± 11	0.89 ± 0.001
GP03O	1.21 ± 0.02	176.9 ± 9·10^6^	1925 ± 51	0.92 ± 0.002
PP01O	1.35 ± 0.01	271.8 ± 7·10^6^	1363 ± 13	0.85 ± 0.001

**Table 6:** Air permeability and velocity of the filter media at 200 Pa.

Sample	Mean Velocity $\bar{v}$ in m/s	Air Permeability B in 10^−12^ m^2^
CP01O	492.8 ± 4.2	55.3 ± 0.5
GC01F	17.7 ± 0.2	1.5 ± 0.0
GC02O	233.9 ± 4.6	26.5 ± 0.5
GP03O	517.5 ± 14.3	56.1 ± 1.6

**Table 7:** Influence of repeated stress on the volume reduction at compression of material sample CP01O.

Cycle	Volume Reduction in %	Plastic Component in %	Elastic Component in %	Viscous Volume Increase in %
1	13.5	5	8.5	
				2.2
2	11.2	2.6	8.6	
				2.1
3	10.8	2.3	8.5	
				2.0
4	10.6	2.1	8.5	
				1.8
5	10.4	2.0	8.4	

**Table 8:** Maximum compressibility and compressibility index (CP01O).

Cycle	Compressibility κ in 1/Pa (at 46 kPa)	Compressibility Index n
1	0.293	0.0267
2	0.242	0.0224
3	0.234	0.0218
4	0.230	0.0215
5	0.225	0.0213

**Table 9:** Young’s modulus $Y^{*}$ of filter media measured by tensile tests in md and cd.

Sample	Breaking Stress in MPa		Young’s Modulus in MPa	
	md	cd	md	cd
CP01O	7.30 ± 0.18	4.73 ± 0.28	142.75 ± 2.18	61.38 ± 2.78
GC01F	13.57 ± 0.15	7.26 ± 0.45	220.33 ± 1.17	94.55 ± 2.07
GC02O	6.89 ± 0.11	3.38 ± 0.17	142.88 ± 2.36	83.48 ± 1.71
GP03O	not reached	1.28 ± 0.17	44.57 ± 8.35	16.14 ± 11.55
PP01O	8.86 ± 1.22	6.35 ± 0.12	91.73 ± 6.48	66.25 ± 3.03

**Table 10:** Poisson’s number ν of filter media measured by tensile tests in md and cd.

Sample	Poisson’s Number (Dimensionless)	
	md	cd
CP01O	0.203	0.186
GC01F	0.178	0.178
GC02O	0.214	0.102

**Table 11:** Changes in the sample thickness after aging in heated oil.

Sample	Increase in Thickness in %
GC01F	55
GP03O	42
PP01O	73

**Table 12:** Young’s modulus after inner and outer aging.

Sample	Young’s Modulus in MPa			
	After Inner Aging		After Outer Aging	
	md	cd	md	cd
GC01F	95 ± 1.1	85 ± 0.7	59 ± 0.4	52 ± 0.6
GP03O	44 ± 1.6	28 ± 2.1	21 ± 5.8	13 ± 1.2
PP01O	52 ± 1.3	42 ± 1.4	25 ± 0.9	21 ± 1.7

**Table 13:** Ratio of Young’s modulus’s initial and aged values.

Sample	Inner Aging in %		Outer Aging in %	
	md	cd	md	cd
GC01F	−57	−10	−73	−45.
GP03O	−1	73	-53	−19
PP01O	−43	−37	−73	−68

**Table 14:** Storage and loss modulus of fuel filter medium samples measured by DMTA.

Sample	Temperature in °C	Orientation	Storage Modulus E′ in MPa	Loss Modulus E″ in MPa
CP01O	20	md	490.9 ± 5.3	13.6 ± 0.7
		cd	231.4 ± 3.0	6.7 ± 0.3
	100	md	418.1 ± 15.0	16.9 ± 1.5
		cd	199.5 ± 9.6	8.4 ± 0.7
GC01F	20	md	1108.6 ± 12.4	33.3 ± 2.8
		cd	489.5 ± 11.6	16.0 ± 0.8
	60	md	1098.9 ± 20.7	34.0 ± 1.3
		cd	478.4 ± 12.6	15.3 ± 0.8
GP03O	20	md	150.8 ± 8.1	22.4 ± 3.7
		cd	77.4 ± 4.4	9.7 ± 1.5
	100	md	126.9 ± 11.3	17.4 ± 1.1
		cd	49.2 ± 3.5	7.0 ± 0.5

## Data Availability

The calculated values of characteristic and mechanical properties are listed in Table 4, Table 5, Table 6, Table 7, Table 8, Table 9, Table 10, Table 11, Table 12, Table 13 and Table 14. The measured data (Figure 7, Figure 8, Figure 9, Figure 12, Figure 13 and Figure 14) can be requested from the authors.
